# Election

**Published:** 1856-01

**Authors:** 


### ELECTION.

At the stated period for the election of officers of this institution, the following were chosen by ballot:

Prof. D. L. M'Gugin, M. D., *President.*

Prof. J. C. Hughes, M. D., *Treasurer;* and Prof. John R. Allen, M. D., *Dean.*

A resolution, cordially approving the conduct and management of the affairs of this institution by Prof. Hughes as Dean, passed unanimously.

